# A simple and efficient protocol for generating transgenic hairy roots using *Agrobacterium rhizogenes*

**DOI:** 10.1371/journal.pone.0291680

**Published:** 2023-11-01

**Authors:** Shaun Ferguson, Nikolaj B. Abel, Dugald Reid, Lene H. Madsen, Thi-Bich Luu, Kasper R. Andersen, Jens Stougaard, Simona Radutoiu

**Affiliations:** 1 Department of Molecular Biology and Genetics, Aarhus University, Aarhus, Denmark; 2 Department of Animal, Plant and Soil Sciences, School of Agriculture, Biomedicine and Environment, La Trobe University, Melbourne, Australia; Nuclear Science and Technology Research Institute, ISLAMIC REPUBLIC OF IRAN

## Abstract

For decades, *Agrobacterium rhizogenes* (now *Rhizobium rhizogenes*), the causative agent of hairy root disease, has been harnessed as an interkingdom DNA delivery tool for generating transgenic hairy roots on a wide variety of plants. One of the strategies involves the construction of transconjugant *R*. *rhizogenes* by transferring gene(s) of interest into previously constructed *R*. *rhizogenes* pBR322 acceptor strains; little has been done, however, to improve upon this system since its implementation. We developed a simplified method utilising bi-parental mating in conjunction with effective counterselection for generating *R*. *rhizogenes* transconjugants. Central to this was the construction of a new Modular Cloning (MoClo) compatible pBR322-derived integration vector (pIV101). Although this protocol remains limited to pBR322 acceptor strains, pIV101 facilitated an efficient construction of recombinant vectors, effective screening of transconjugants, and RP4-based mobilisation compatibility that enabled simplified conjugal transfer. Transconjugants from this system were tested on *Lotus japonicus* and found to be efficient for the transformation of transgenic hairy roots and supported infection of nodules by a rhizobia symbiont. The expedited protocol detailed herein substantially decreased both the time and labour for creating transconjugant *R*. *rhizogenes* for the subsequent transgenic hairy root transformation of *Lotus*, and it could readily be applied for the transformation of other plants.

## Introduction

Hairy root disease occurs when a plant becomes infected by *Rhizobium rhizogenes* (previously known as *Agrobacterium rhizogenes* [1]), which causes neoplastic growth at the site of infection, resulting in highly branched roots [2]. *R*. *rhizogenes* can induce hairy roots on a diverse range of monocotyledonous and dicotyledonous plants, which become susceptible when wounded [3–5]. A distinct phenolic compound profile is produced by damaged plant cells which activates a set of virulence (*vir*) genes resulting in the transfer of segments of DNA, known as transfer-DNA (T-DNA), from the root-inducing plasmid (pRi) to the plant host [5]. The T-DNA segment is demarcated by 25 bp repeat sequences, the only c*is*-acting requirement for transfer [6]. Following the discovery of this natural interkingdom DNA transfer mechanism, various strategies have emerged for generating transgenic hairy roots [7–10] for a plethora of applications, including, but not limited to: molecular breeding, metabolic engineering, recombinant protein production, rhizosphere physiology, and root physiology (e.g. nitrogen fixation) [11, 12].

One hairy root transformation approach uses ‘binary vectors’ that encode a segment of T-DNA carrying a gene of interest to be transferred to the plant. A range of binary vector options are available [13], and that system provides a simple method for constructing *R*. *rhizogenes* transconjugants, often directly via electroporation. Generally, those transconjugants maintain the binary vector alongside pRi, enabling co-transformation of both the native pRi T-DNA and the foreign DNA within the binary vector T-DNA. The binary vector system is appealing due to its simplicity and convenience, yet it is subject to considerable variation in co-transformation frequency, which can often result in poor numbers of transgenic hairy roots [10, 14, 15].

An alternative strategy relies on the introduction of suicide vectors into *R*. *rhizogenes* and two variants of this strategy can be implemented: integration vectors that encode a restriction fragment of the pRi TL-DNA, or pBR322-derived plasmids that can be used with previously constructed *R*. *rhizogenes* pBR322 acceptor strains. *R*. *rhizogenes* pBR322 acceptor strains were constructed by integrating pBR322-derived plasmids into the pRi15834 TL-DNA region by replacement mutagenesis [10, 16]. Both variants function by integrating the suicide vector into the pRi TL-DNA of *R*. *rhizogenes* through homologous recombination. The *R*. *rhizogenes* strain AR1193 is a pBR322 acceptor strain [10] that was developed for hairy root production in *Lotus corniculatus* [17] and its diploid relative *Lotus japonicus*. AR1193 has since successfully generated hairy roots in a range of plants, including significant species such as *Pisum sativum* (pea), *Brassica napus* (rapeseed), *Phtheirospermum japonicum* (Phtheirospermum), and *Medicago truncatula* (Barrel medic) [18–21]. Despite this, the main problem with the integration strategies stems from the extended workload and resources necessary to construct transconjugant *R*. *rhizogenes*. The major advantage, however, is the consistent and high rate of transgenic hairy roots [10].

Until now, the pBR322-derived vector pIV10 was utilised to transform *R*. *rhizogenes* pBR322 acceptor strains [14]. The use of pIV10 is reliant on *E*. *coli* strain GJ23, which contains two helper plasmids, R64drdl1 and pGJ28, that encode transfer (*tra*) and ColE1 mobilisation functions, respectively [22]. This out-dated transfer system requiring helper strain GJ23 for transfer of pIV10 is a major contributing factor to the unnecessary workload of this method.

Therefore, the purpose of this study was to create a simplified and streamlined protocol for generating transconjugant *R*. *rhizogenes* via the pBR322 acceptor-based strategy. We constructed a modified version of pIV10, resulting in the new vector pIV101. Crucially, pIV101 now contains an IncP *oirT* compatible with RP4 transfer machinery and, additionally, the *lacZα* fragment situated within a Golden Gate Assembly (GGA) compatible integration site [23, 24]. The addition of RP4 *oriT* renders pIV101 compatible with commonly used *E*. *coli* strains that contain the IncP RP4 transfer machinery integrated into their chromosome [25]. In addition, these strains often carry an auxotrophy for counterselection, e.g. *E*. *coli* ST18 and MFD*pir* [26, 27]. Together, these features provide efficient transfer of compatible mobilisable plasmids to recipient bacteria.

This system was originally developed for *Lotus*, as it is a model legume from the *Leguminosae* family. Many legumes are capable of forming a symbiotic partnership with bacteria known as rhizobia, where they become housed in structures on the roots, called nodules, and they fix atmospheric nitrogen for the plant to utilise as a nitrogen source [28]. *Lotus* has provided extensive understanding of legume–rhizobia symbiosis, in part due to the potential for generating transgenic plants through transformation with *R*. *rhizogenes* [29, 30]. Accordingly, to test the efficacy of pIV101, we investigated hairy root transformation of *Lotus japonicus* using *R*. *rhizogenes* AR1193 and our new plasmid.

We directly compared the original pIV10-based method with the new protocol utilising vector pIV101, to construct transconjugant *R*. *rhizogenes* AR1193. The time and labour required was markedly reduced when using pIV101, and critically, when we infected *Lotus* with transconjugants generated by the parallel methodologies, we observed equal efficiencies for both transgenic root transformation and the ability to form infected nodules when subsequently inoculated with rhizobia.

## Materials and methods

The protocol described in this peer-reviewed article is published on protocols.io, (DOI: dx.doi.org/10.17504/protocols.io.261ge3xkjl47/v1) and is included for printing as S1 File with this article.

## Results

### Construction of pIV101, an improved pBR322-derived Golden Gate Assembly destination plasmid for transformation of *R*. *rhizogenes*

The original4.5 kb pIV10 was a composite of several plasmids, including the pBR322 sequence encoding the ColE1 origin of replication, the ColE1 basis of motility (*bom*), and ampicillin resistance from plasmid pHC79, the spectinomycin/streptomycin resistance from plasmid R702, and the pUC19 EcoRI/HindIII polylinker [10, 14, 31]. The pBR322 *bom* site enables *in trans* mobilisation of the plasmid by the ColE1 mobilisation functions, and the ColE1 *oriV* capacitates replication in *E*. *coli* but not in *R*. *rhizogenes*, making it a suicide plasmid in that species [32]. Transfer of exogenous DNA via conjugation into *R*. *rhizogenes* with pIV10 requires *E*. *coli* strain GJ23, which contains two helper plasmids, R64drdl1 and pGJ28, that encode transfer (*tra*) and ColE1 mobilisation functions, respectively [22].

**Fig 1:**
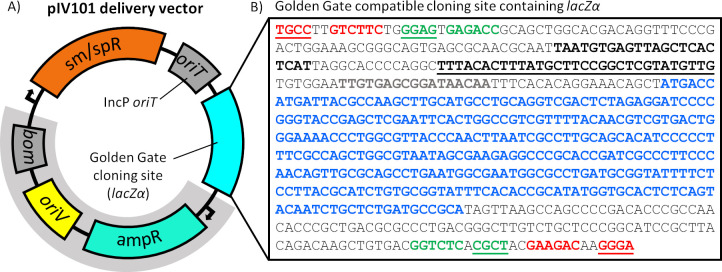
pBR322-derived *R*. *rhizogenes* transformation vector pIV101. A) Schematic representation of pIV101 indicating: the *cis* acting ColE1 basis of mobility (*bom*), pBR322 vegetative origin of replication (*oriV*), ampicillin resistance (ampR), spectinomycin/streptomycin resistance (sm/spR), the *cis* acting IncP origin of transfer (*oriT*), the Golden Gate Assembly cloning site (cyan box), and 2,297 bp of the original pBR322 plasmid sequence (highlighted in grey). B) Sequence of the Golden Gate Assembly cloning site with important features bolded: the bpiI recognition sequences (red) with corresponding fusion sites (red/underlined), the bsaI recognition sequences (green) with corresponding fusion sites (green/underlined), the *lacZα* fragment (blue), the CAP binding site (black), the *lac* promoter (black/underlined), and the *lac* operator (grey).

We made two important modifications to improve pIV10, resulting in the new vector pIV101. First, the pUC19 polylinker sequence was replaced with a GGA compatible cloning site and domesticated for BpiI and BsaI recognition sequences to implement the Modular Cloning (MoClo) system developed by Weber *et al*. [33]. Secondly, we included a new IncP origin of transfer. Within pIV10, there is a section of 2,297 bp of pBR322 sequence (Fig 1A) that must remain unmodified, as it is required for homologous recombination with pBR322 sequence integrated within the TL-DNA of the *R*. *rhizogenes* acceptor strains [16]. Therefore, in pIV101 the original ColE1 *bom* remains and the additional IncP *oriT* was inserted between the GGA cloning site and the sp/sm resistance genes to enable mobilisation with RP4 *tra* functions (Fig 1A) without affecting recombination capability.

We designed pIV101 to be a level 2 destination vector (pL2-1) based on the MoClo GGA strategy [33]; as such, the GGA cloning site contains TGCC and GGGA fusion sites generated by digestion with BpiI. Additionally, the GGA cloning site contains nested BsaI recognition sequences, which upon digestion will result in GGAG and CGCT fusion sites compatible with the insertion of level 1 fragments (Fig 1B). The *lacZα* fragment was incorporated within the nested restriction sites of the GGA cloning site to permit blue/white screening (Fig 1B).

### Optimised workflow for construction of transconjugant *R*. *rhizogenes* clones

**Fig 2:**
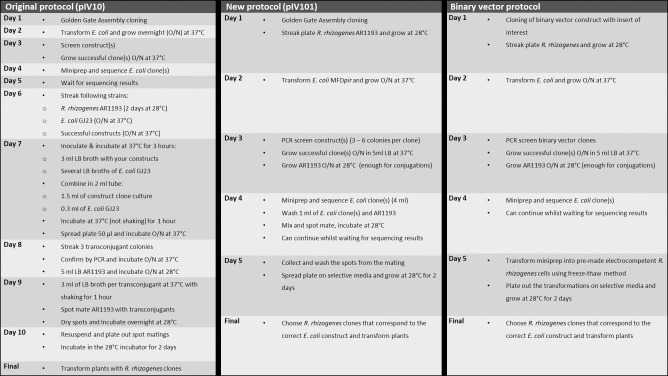
Simplified overview comparing several workflows required to construct transconjugant *R*. *rhizogenes* for subsequent hairy root transformation. The left column represents the protocol followed when using pBR322-derived vector pIV10; the middle column represents an optimised and simplified version of the pBR322-derived vector protocol using pIV101; the right column represents a protocol for transformation using a binary vector.

The generation of transconjugant *R*. *rhizogenes* previously required a minimum of 12 days with substantial hands-on time. By utilising the modified pBR322-derived level 2 destination vector (pIV101), it was possible to construct transconjugant *R*. *rhizogenes* in seven days, with less hands-on time, a substantial decrease in time and labour comparable to that required for a binary vector transformation system (Fig 2).

### Transgenic hairy root transformation of *Lotus* remains equally efficient with pIV101

To compare pIV101 to the original pIV10 vector, an insert was cloned into each vector and subsequently used for hairy root transformation of wild-type *Lotus japonicus* Gifu in parallel.

A separate GGA was performed for each destination vector in a reaction that included the preassembled level 1 vector (PMC-03820) (Table 1) encoding the *Lotus* ubiquitin promoter (pUBI) driving nuclear-localised triple YFP (tYFP) followed by a 35s terminator. This was combined with the level 1 end-linker with either *lacZα* (pL1M-ELB-1-49255) or the level 1 end-linker empty (pL1M-ELE-1-41722) to assemble the level 2 constructs, pIV10::pUBI-tYFP and pIV101::pUBI-tYFP (Table 1), respectively. For pIV10, the GGA reaction included the end-linker containing *lacZα* for blue/white selection. For pIV101, the empty end-linker was substituted, as *lacZα* is present within the GGA cloning site.

**Table 1:** Strains and plasmids used in this study.

Strains	Genotypes/features	Reference
*Escherichia coli*		
GJ23	*E*. *coli* JC2926 (*recA* derivative of ABI157) + pGJ28 + R64*drd*l1	[22]
TOP10	F- *mcrA* Δ(*mrr-hsdRMS-mcrBC*) φ80*lacZ*ΔM15 Δ*lacX74 nupG recA1 araD139* Δ(*ara leu*)7697 *galE15 galK16 rpsL*(Str^r^) *endA1* λ^-^	Invitrogen
ST18	S17 λ*pir*Δ*hemA*	[27]
MFD*pir*	MG1655 RP4-2-Tc::[ΔMu1::*aac(3)IV*-Δ*aphA*-Δ*nic*35-ΔMu2::*zeo*] Δ*dapA*::(*erm-pir*) Δ*recA*	[26]
*Agrobacterium rhizogenes*		
C58CI pRi15834	Rif^r^, wild-type	Max-Planck, Cologne/[16]
AR1193	pRi1193 carrying pBR322 in the TL segment	[10]
*Mesorhizobium japonicum*		
MAF303099	Wild-type strains carrying chromosomal DsRed insertion constitutively expressed	[34]
Plasmids		
pGJ28	Km/Nm^r^ Cda^+^ Ida^+^ ColD replicon carrying ColEl *mob* and *bom*	[22]
R64*drd*l1	Tc^r^ Sm^r^ Iα-type plasmid, transfer-derepressed derivative of R64	[22]
pIV10	*ori*pBR322, ColEl *mob* and *bom* Sm^r^/Sp^r^ Ap^r^	[31]
pIV101	*ori*pBR322, ColEl *mob* and *bom* RP4-*oriT* Smr/Sp^r^ Ap^r^ GGA cloning site containing *lacZα*	This study, Addgene plasmid #196671
PMC-03820	Level 1 vector containing the *Lotus* ubiquitin promoter (pUBI), triple YFP (tYFP-NLS), and 35s terminator, Ap^r^	This study
pL1M-ELB-1-49255	For end linking after pos-1 in a Level 2i-1 (multi gene) construct containing *lacZα*, Ap^r^	This study
pL1M-ELE-1-41722	For end linking after pos-1 in a Level 2 (multi gene) construct, Ap^r^	This study
pIV10::pUBI-tYFP	Level 2 construct pIV10 carrying tYFP expressed by *Lotus* ubiquitin promoter	This study
pIV101::pUBI-tYFP	Level 2 construct pIV101 carrying tYFP expressed by *Lotus* ubiquitin promoter	This study

**Fig 3:**
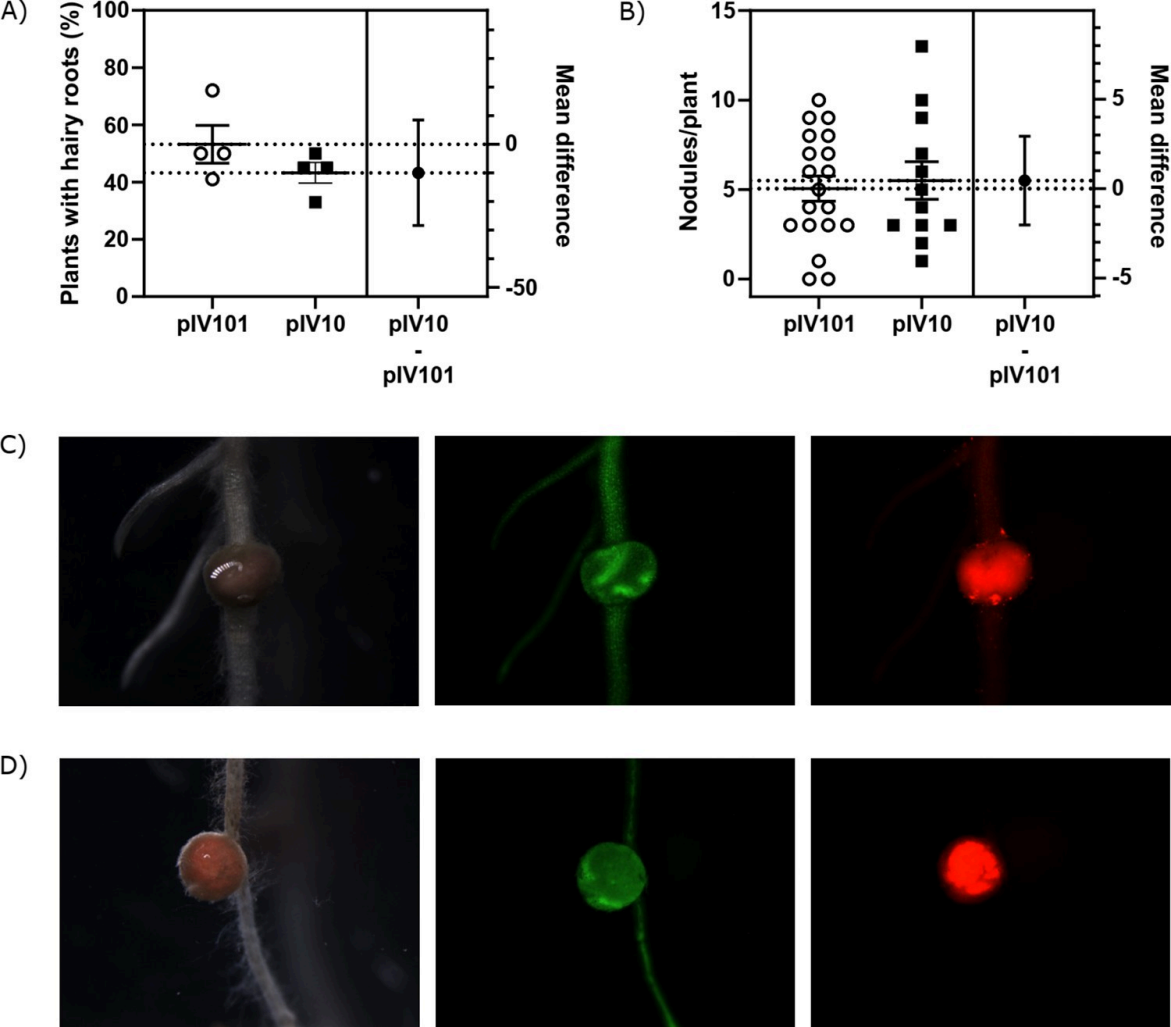
*R*. *rhizogenes* mediated hairy root formation on *L*. *japonicus* Gifu was unaffected by the plasmid used for transformation. (A) The number of hairy roots formed three weeks post infection by *R*. *rhizogenes* clones transformed by either pIV10 or pIV101, each containing pUBI-tYFP. Each symbol represents the average number of plants with hairy roots from one replicate, and the error bars represent ± the SEM. (B) The number of nodules formed on the same hairy roots from (A), three weeks post inoculation with *Mesorhizobium japoniucm* MAF303099. Each symbol represents a single plant, and the error bars represent ± the SEM. Unpaired *t*-tests for (A) and (B) revealed no significant difference between the two plasmid groups as displayed on the right *y*-axis of each graph, which represents the 95% CI. (C) and (D) Nodules formed by *M*. *japonicum* expressing DsRed on hairy roots induced by *R*. *rhizogenes* that was transformed by either pIV101 (C) or pIV10 (D). Both plasmids enabled similar expression of tYFP and resulted in infected nodules.

Afterwards, pIV10::pUBI-tYFP and pIV101::pUBI-tYFP were transformed into *E*. *coli* MFD*pir* and conjugated into *R*. *rhizogenes* AR1193 as described in Fig 2 and Materials and Methods. Hairy root transformation was performed on 45 and 46 plants with pIV101::pUBI-tYFP and pIV10::pUBI-tYFP, respectively, divided into four replica. A similar percentage of plants showed the formation of hairy roots after three weeks (53% and 43%, respectively) (Fig 3A).

To validate whether the hairy roots induced by *R*. *rhizogenes* transformed with pIV101 remain efficacious for nodule formation by symbiotic rhizobia, we inoculated plants with *Mesorhizobium japonicum* expressing the DsRed protein [34]. Hairy roots induced by *R*. *rhizogenes* transformed with either construct formed infected nodules to a similar level three weeks post inoculation (Fig 3B) and revealed similar morphology (Fig 3C and 3D).

Therefore, we successfully expedited the construction of transconjugant *R*. *rhizogenes* clones in order to make transgenic hairy roots, without affecting the effectiveness of hairy root formation, nodule infection by rhizobia, or morphology of the nodule. Furthermore, we have no reason to believe pIV101 would not work similarly in studies where other plant–microbe interactions are investigated, provided an appropriate *R*. *rhizogenes* pBR322-acceptor strain is available or could be constructed. Thus, we believe that this vector will be highly beneficial for plant studies across a range of fields.

## Supporting information

S1 File Step-by-step protocol, also available on protocols.io. (PDF)

## References

[1] Young J, Kuykendall L, Martinez-Romero E, Kerr A, Sawada H. A revision of Rhizobium Frank 1889, with an emended description of the genus, and the inclusion of all species of Agrobacterium Conn 1942 and Allorhizobium undicola de Lajudie et al. 1998 as new combinations: *Rhizobium radiobacter*, *R*. *rhizogenes*, *R*. *rubi*, *R*. *undicola* and *R*. *vitis*. International Journal of Systematic and Evolutionary Microbiology. 2001;51(1):89–103. 10.1099/00207713-51-1-89. 11211278

[2] Chilton M-D, Tepfer DA, Petit A, David C, Casse-Delbart F, Tempé J. *Agrobacterium rhizogenes* inserts T-DNA into the genomes of the host plant root cells. Nature. 1982;295(5848):432–4. doi: 10.1080/15592324.2022.2097469

[3] Christey MC. Use of Ri-mediated transformation for production of transgenic plants. In Vitro Cellular & Developmental Biology-Plant. 2001;37(6):687–700. doi: 10.1007/s11627-001-0120-0

[4] Ono NN, Tian L. The multiplicity of hairy root cultures: prolific possibilities. Plant Science. 2011;180(3):439–46. doi: 10.1016/j.plantsci.2010.11.012 21421390

[5] Zambryski P, Tempe J, Schell J. Transfer and function of T-DNA genes from *Agrobacterium* Ti and Ri plasmids in plants. Cell. 1989;56(2):193–201. 10.1016/0092-8674(89)90892-1. 2643473

[6] Bouchez D, Tourneur J. Organization of the agropine synthesis region of the T-DNA of the Ri plasmid from *Agrobacterium rhizogenes*. Plasmid. 1991;25(1):27–39. 10.1016/0147-619X(91)90004-G. 1852015

[7] Kloppholz S, Kuhn H, Requena N. A secreted fungal effector of *Glomus intraradices* promotes symbiotic biotrophy. Current Biology. 2011;21(14):1204–9. 10.1016/j.cub.2011.06.044. 21757354

[8] Petit A, Stougaard J, Kühle A, Marcker KA, Tempé J. Transformation and regeneration of the legume *Lotus corniculatus*: a system for molecular studies of symbiotic nitrogen fixation. Molecular and General Genetics MGG. 1987;207(2):245–50. 10.1007/BF00331585.

[9] Stougaard J. *Agobacterium rhizogenes* as a vector for transforming higher plants. Plant Gene Transfer and Expression Protocols. 1995:49–61. doi: 10.1385/0-89603-321-X:49. 8563829

[10] Stougaard J, Abildsten D, Marcker KA. The *Agrobacterium rhizogenes* pRi TL-DNA segment as a gene vector system for transformation of plants. Molecular and General Genetics MGG. 1987;207(2):251–5. 10.1007/BF00331586.

[11] Gutierrez-Valdes N, Häkkinen ST, Lemasson C, Guillet M, Oksman-Caldentey K-M, Ritala A, et al. Hairy root cultures—a versatile tool with multiple applications. Frontiers in plant science. 2020;11:33. doi: 10.3389/fpls.2020.00033 32194578 PMC7064051

[12] Oresnik IJ, Pacarynuk LA, O’Brien SA, Yost CK, Hynes MF. Plasmid-encoded catabolic genes in *Rhizobium leguminosarum* bv. *trifolii*: evidence for a plant-inducible rhamnose locus involved in competition for nodulation. Molecular Plant-Microbe Interactions. 1998;11(12):1175–85.

[13] Bahramnejad B, Naji M, Bose R, Jha S. A critical review on use of *Agrobacterium rhizogenes* and their associated binary vectors for plant transformation. Biotechnology Advances. 2019;37(7):107405. 10.1016/j.biotechadv.2019.06.004. 31185263

[14] Radutoiu S, Madsen LH, Madsen EB, Nielsen AM, Stougaard J. *Agrobacterium rhizogenes* pRi TL-DNA integration system: a gene vector for Lotus japonicus transformation. *Lotus japonicus* Handbook: Springer; 2005. p. 285–7.

[15] Wang Y, Yang F, Zhu P-F, Khan A, Xie Z-P, Staehelin C. Use of the rhizobial type III effector gene *nopP* to improve *Agrobacterium rhizogenes*-mediated transformation of *Lotus japonicus*. Plant Methods. 2021;17(1):1–11. doi: 10.1186/s13007-021-00764-z 34162409 PMC8220826

[16] Hansen J, Jørgensen J-E, Stougaard J, Marcker KA. Hairy roots—a short cut to transgenic root nodules. Plant Cell Reports. 1989;8(1):12–5. doi: 10.1007/BF00735768 24232586

[17] Jensen JS, Marcker KA, Otten L, Schell J. Nodule-specific expression of a chimaeric soybean leghaemoglobin gene in transgenic *Lotus corniculatus*. Nature. 1986;321(6071):669–74. doi: 10.1038/321669a0

[18] Clemow SR, Clairmont L, Madsen LH, Guinel FC. Reproducible hairy root transformation and spot-inoculation methods to study root symbioses of pea. Plant Methods. 2011;7(1):1–15. doi: 10.1186/1746-4811-7-46 22172023 PMC3264533

[19] Damgaard O, Rasmussen O. Direct regeneration of transformed shoots in *Brassica napus* from hypocotyl infections with *Agrobacterium rhizogenes*. Plant molecular biology. 1991;17:1–8. doi: 10.1007/bf00036800 1651126

[20] Geddes BA, Paramasivan P, Joffrin A, Thompson AL, Christensen K, Jorrin B, et al. Engineering transkingdom signalling in plants to control gene expression in rhizosphere bacteria. Nature communications. 2019;10(1):3430. doi: 10.1038/s41467-019-10882-x 31366919 PMC6668481

[21] Ishida JK, Yoshida S, Ito M, Namba S, Shirasu K. *Agrobacterium rhizogenes*-mediated transformation of the parasitic plant *Phtheirospermum japonicum*. PloS one. 2011;6(10):e25802. doi: 10.1371/journal.pone.0025802 21991355 PMC3185032

[22] Van Haute E, Joos H, Maes M, Warren G, Van Montagu M, Schell J. Intergeneric transfer and exchange recombination of restriction fragments cloned in pBR322: a novel strategy for the reversed genetics of the Ti plasmids of *Agrobacterium tumefaciens*. The EMBO Journal. 1983;2(3):411–7. 10.1002/j.1460-2075.1983.tb01438.x. 11894957 PMC555148

[23] Engler C, Gruetzner R, Kandzia R, Marillonnet S. Golden gate shuffling: a one-pot DNA shuffling method based on type IIs restriction enzymes. PloS One. 2009;4(5):e5553. doi: 10.1371/journal.pone.0005553 19436741 PMC2677662

[24] Engler C, Kandzia R, Marillonnet S. A one pot, one step, precision cloning method with high throughput capability. PloS One. 2008;3(11):e3647. doi: 10.1371/journal.pone.0003647 18985154 PMC2574415

[25] Simon R, Priefer U, Pühler A. A broad host range mobilization system for in vivo genetic engineering: transposon mutagenesis in gram negative bacteria. Bio/technology. 1983;1(9):784–91. 10.1038/nbt1183-784.

[26] Ferrieres L, Hémery G, Nham T, Guérout A-M, Mazel D, Beloin C, et al. Silent mischief: bacteriophage Mu insertions contaminate products of *Escherichia coli* random mutagenesis performed using suicidal transposon delivery plasmids mobilized by broad-host-range RP4 conjugative machinery. Journal of Bacteriology. 2010;192(24):6418–27. 10.1128/JB.00621-10. 20935093 PMC3008518

[27] Thoma S, Schobert M. An improved *Escherichia coli* donor strain for diparental mating. FEMS Microbiology Letters. 2009;294(2):127–32. 10.1111/j.1574-6968.2009.01556.x. 19431232

[28] Oldroyd GE. Speak, friend, and enter: signalling systems that promote beneficial symbiotic associations in plants. Nature Reviews: Microbiology. 2013;11(4):252. doi: 10.1038/nrmicro2990 23493145

[29] Handberg K, Stougaard J. *Lotus japonicus*, an autogamous, diploid legume species for classical and molecular genetics. The Plant Journal. 1992;2(4):487–96. 10.1111/j.1365-313X.1992.00487.x.

[30] Stougaard J. *Lotus japonicus*: A Model Plant for the Legume Family. Reference Module in Life Sciences. 2017.

[31] Ramlov KB, Laursen NB, Stougaard J, Marcker KA. Site‐directed mutagenesis of the organ‐specific element in the soybean leghemoglobin Ibc3 gene promoter. The Plant Journal. 1993;4(3):577–80. 10.1046/j.1365-313X.1993.04030577.x. 8220496

[32] Zambryski P, Joos H, Genetello C, Leemans J, Van Montagu M, Schell J. Ti plasmid vector for the introduction of DNA into plant cells without alteration of their normal regeneration capacity. The EMBO Journal. 1983;2(12):2143–50. doi: 10.1002/j.1460-2075.1983.tb01715.x 16453482 PMC555426

[33] Weber E, Engler C, Gruetzner R, Werner S, Marillonnet S. A modular cloning system for standardized assembly of multigene constructs. PloS One. 2011;6(2):e16765. doi: 10.1371/journal.pone.0016765 21364738 PMC3041749

[34] Maekawa T, Maekawa‐Yoshikawa M, Takeda N, Imaizumi‐Anraku H, Murooka Y, Hayashi M. Gibberellin controls the nodulation signaling pathway in *Lotus japonicus*. The Plant Journal. 2009;58(2):183–94. 10.1111/j.1365-313X.2008.03774.x. 19121107

