# Supplementary material for: A simple and efficient protocol for generating transgenic hairy roots using Agrobacterium rhizogenes
Source: PLoS One. 2023 Nov 1;18(11):e0291680. doi: 10.1371/journal.pone.0291680 (PMC10619795; doi:10.1371/journal.pone.0291680)
Supplement: S1 File — (PDF) [file pone.0291680.s001.pdf]

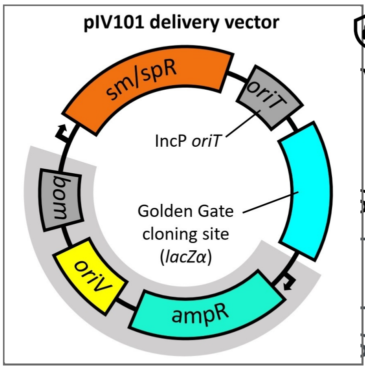

A simple and efficient protocol for generating transgenic hairy roots using *Agrobacterium rhizogenes*

Shaun Ferguson[1], Nikolaj B. Abel[1], Dugald Reid[2], Lene H. Madsen[1], Thi-Bich Luu[1], Kasper R. Andersen[1], Jens Stougaard[1], Simona Radutoiu[1]

[1]Department of Molecular Biology and Genetics, Aarhus University, Denmark; [2]Department of Animal, Plant and Soil Sciences, School of Agriculture, Biomedicine and Environment, La Trobe University, Melbourne, Australia

Shaun Ferguson
Department of Molecular Biology and Genetics, Aarhus Univers...

Protocol Info: Shaun Ferguson, Nikolaj B. Abel, Dugald Reid, Lene H. Madsen, Thi-Bich Luu, Kasper R. Andersen, Jens Stougaard, Simona Radutoiu . A simple and efficient protocol for generating transgenic hairy roots using Agrobacterium rhizogenes. **protocols.io** https://protocols.io/view/a-simple-and-efficient-protocol-for-generating-tra-cnbnvame

Created: Jan 25, 2023

Last Modified: Aug 21, 2023

PROTOCOL integer ID: 75854

ABSTRACT

For decades, *Agrobacterium rhizogenes* (now *Rhizobium rhizogenes*), the causative agent of hairy root disease, has been harnessed as an interkingdom DNA delivery tool for generating transgenic hairy roots on a wide variety of plants. One of the strategies involves the construction of transconjugant *R. rhizogenes* by transferring gene(s) of interest into previously constructed *R. rhizogenes* pBR322 acceptor strains; little has been done, however, to improve upon this system since its implementation. We developed a simplified method utilising bi-parental mating in conjunction with effective counterselection for generating *R. rhizogenes* transconjugants. Central to this was the construction of a new Modular Cloning (MoClo) compatible pBR322-derived integration vector (pIV101). Although this protocol remains limited to pBR322 acceptor strains, pIV101 facilitated an efficient construction of recombinant vectors, effective screening of transconjugants, and RP4-based mobilisation compatibility that enabled simplified conjugal transfer.Transconjugants from this system were tested on *Lotus japonicus* and found to be efficient for the transformation of transgenic hairy roots and supported infection of nodules by a rhizobia symbiont. The expedited protocol detailed herein substantially decreased both the time and labour for creating transconjugant *R. rhizogenes* for the subsequent transgenic hairy root transformation of *Lotus*, and it could readily be applied for the transformation of other plants.

GUIDELINES

The Modular Cloning (MoClo) strategy implemented for pIV101 is based on the publication by Weber *et al.* 2011.

For this protocol we use *E. coli* strain ST18 for transformation of our cloned construct and subsequent biparental mating to transfer the construct to *R. rhizogenes.* Another *E. coli* strain could be substituted but it must contain the RP4 conjugative machinery for mobilisation of pIV101 and we would recommend that it also contains the *lacZ*ΔM15 mutation for blue white/screening as well as an auxotrophy for efficicient conterselection (E.g. Δ*dapA*).

Ensure you wash the cells before and after biparental mating. This ensures adequate removal of both the antibiotics for plasmid selection, and the supplements required by the auxotrophic *E. coli.*

For standard molecular biology techniques (E.g. PCR, plasmid preparation etc.) please refer to Molecular Cloning: A Laboratory Manual, 4th edition.

MATERIALS

Chemicals for Golden Gate reaction

- Vector backbone (pIV101)
- Additional assembly piece(s)
- 10X NEB BSA (If using BsaI-HF®v2)
- NEB BsaI-HF®v2
- NEB BbsI-HF®
- 10X NEB T4 Ligase Buffer
- NEB T4 Ligase, 2,000,000 cohesive end units/ml
- Nuclease-free water

Chemicals for *E. coli* chemical transformation

- Filter sterilised diaminopimelic acid (DAP) stock at 30 mM in water (For Δ*dapA* mutant *E. coli* strains).
- Previously prepared chemically competent aliquots of *E. coli* donor strain.
- KCM buffer (5X)
- 5-Bromo-4-chloro-3-indolyl β-D-galactopyranoside (X-gal) 1:1000

Media for bacteria

- LB medium

Material for seed germination and hairy root transformation

- Sand paper
- Hypochlorite (12% stock solution)
- Nuclease-free water
- Filter paper

- Gamborg B5 media including vitamins (Duchefa-biochemie)
- Scalpel for plant cutting
- Syringe needles (0.4 mm)
- Parafilm
- 3M Milipore tape

Antibiotics(Stock)
- Rifampicin (50 mg/ml DMSO)
- Streptomycin (50 mg/ml water)
- Spectinomycin (50 mg/ml water)
- Ampicillin (100 mg/ml water)

Buffers and stock solutions

KCM Buffer (5X):
- 0.5M KCl
- 0.15 M $CaCl_2$
- 0.25 M $MgCl_2$
- Filter sterilise through 0.2 uM filter. Store at -20.

5-Bromo-4-chloro-3-indolyl β-D-galactopyranoside (X-gal):
- 20 mg/ml in dimethylformamide (2% w/v)

Auxotrophic *E. coli* supplement

5-aminolevulinic acid (ALA):
- 50 mg/ml dissolved in water and filter sterlised

Diaminopemelic acid (DAP):
- 30 mM dissolved in water and filter sterlised

General materials
- Thermocycler suitable for Golden Gate reactions
- Tabletop microcentrifuge
- Spectrophotometer
- 1.5 ml nuclease-free microcentrifuge tubes
- Round and square (11 cm^2) petri dishes

SAFETY WARNINGS

Please refer to the manufacturers safety warnings for the individual reagents and chemicals used throughout.

BEFORE START INSTRUCTIONS

Ensure you have an appropriate *E. coli* cloning/donor strain that contains the RP4 conjugative machinery to enable conjugal transfer of pIV101. Some commonly used strains for this include: *E. coli* S17/ST18, *E. coli* MFD*pir*, *E. coli* WM3064.

If using an auxotrophic *E. coli* strain such as ST18 or MFD*pir* make sure you have the required supplement (e.g. 5-aminolevulinic acid (ALA) for ST18 or diaminopimelic acid (DAP) for MFD*pir*).

pIV101 is available on Addgene and can be found here: https://www.addgene.org/196671/.

Ensure you have a pBR322-derived *R. rhizogenes* acceptor strain e.g. *R. rhizogenes* AR1193.

Golden Gate cloning reaction with plasmid pIV101

1 Add the following components to a Golden Gate reaction: 15m

- Plasmid pIV101 DNA (100 ng) - X µL
- Insert(s) DNA (100 ng) - X µL
- Ligase buffer (10x) - 1 µL
- T4 DNA Ligase - 1 µL
- BpiI (BbsI) (or BsaI for level 1 constructs) - 1 µL
- MilliQ water - up to final volume of 10 µL

1.1 4h

Incubate reaction in thermocycler with the following steps:

Temperature	Time	Cycles
37C	00:05:00	30-60 X
16C	00:05:00	
80C	00:10:00	1

Note

If the Golden Gate reaction is failing to incorporate your desired insert, an additional step that may help:

Following completion of the reaction cycle from step 1.1 - add 1 µl of the restriction enzyme used in the reaction (step 1) to the mix again and incubate for a further 1 - 2hrs at 37C

Because the ligase has already been deactivated, additional active restriction enzyme can help to deplete any remaining plasmids that do not contain the desired insert fragments

Transformation of chemical competent *E. coli* ST18

2 Mix in a 1.5 ml tube: 5m

- 20 µL 5X KCM buffer
- 5 µL Golden Gate cloning mix (from step 1.1 following completion of the program)
- 75 µL ddH_2O

3 Incubate for 00:02:00 on ice 2m

4 Thaw a 100 µL aliquot of chemically competent *E. coli* ST18 cells on ice

5 Mix together the chemical competent *E. coli* cells and the reaction mixture from step 2 by pipetting 1m

6 Incubate the mix for 00:20:00 on ice 20m

7 Incubate for 00:10:00 at Room temperature 10m

8 Add 800 µL LB with 5 µg/ml 5-aminolevulinic acid (ALA) and grow for 01:00:00 at 37 °C with shaking 1h

9 Centrifuge to pellet the cells at 14000 rcf, 25°C, 00:02:00 2m

10 Resuspend the pellet in 100 µL of LB containing 50 µg/ml ALA 2m

11 Plate out the 100 µL of resuspended pellet onto LB agar containing 100 µg/ml ampicillin, 150 µg/ml spectinomycin, and 50 µg/ml ALA 5m

11.1

- If using blue/white selection then also add 2% (w/v) 5-Bromo-4-chloro-3-indolyl β-D-galactopyranoside (X-gal) to the media

12 Grow at 37 °C Overnight and check for colonies the following day 16h

Note

If you are having difficulties obtaining *E. coli* transformants than we suggest trying electrocompetent *E. coli* cells which have a higher transformation efficiency. For transformation by electroporation we recommend first performing a clean up of the Golden Gate reaction mixture DNA to prevent arcing

12.1 If using X-gal for selection avoid blue colonies as pIV101 contains the *lacZα* fragment in the GGA cloning site

12.2 In parallel with the next step (step 13), select colonies from the transformation (step 12) and confirm the construct from the Golden Gate assembly. This can be carried out by several standard approaches: 1d

- Perform colony PCR to amplify the region of the plasmid that contains the GGA cloning site to ensure that the cloning site contains the expected insert size
- This product can be sent for further confirmation by Sanger sequencing
- Additionally, perform a plasmid preparation (miniprep) from the *E. coli* clone which can then be used as template for PCR amplification or for whole plasmid sequencing

> Note
>
> If your insert is large then we recommended sending the construct for whole plasmid sequencing as an alternative to confirmation by standard Sanger sequencing

R. rhizogenes transformation

13 10m

> Note
>
> This section and the next section can be carried out in parallel to save time

Start liquid culture of the wild-type *Agrobacterium rhizogenes* (now *Rhizobium rhizogenes*) from a single colony in LB media with 100 µg/ml rifampicin (a 5 ml broth is sufficient)

14 Incubate for 48:00:00 at 28 °C with shaking 2d

15 Inoculate an LB broth containing 100 µg/ml ampicillin, 150 µg/ml spectinomycin, and 50 µg/ml ALA with a single colony for an ST18 clone carrying the construct of interest (from the previous section) 5m

16 Incubate the LB broth from step 15 Overnight at 37 °C with shaking. 1d

17 Centrifuge 1 ml of the overnight broth for the *E. coli* ST18 carrying the construct of interest at 14000 rcf, 00:02:00 2m

18 Resuspend the *E. coli* pellet in 1 mL sterile dH_2O and repeat the previous step. This is to wash away the broth culture containing antibiotics 3m

19 Resuspend the *E. coli* pellet in 50 µL sterile dH_2O 2m

20 Centrifuge 1 ml of the broth culture of *R. rhizogenes* from step 13-14 at 8000 rcf, 00:02:00 2m

21 Resuspend the *R. rhizogenes* pellet in 1 mL sterile dH_2O and repeat the previous step. This is to wash away the broth culture containing antibiotics 3m

22 Resuspend the *R. rhizogenes* pellet in 50 µL sterile dH_2O 2m

23 Perform a biparental mating by mixing the resuspended *E.coli* ST18 (step 19) and *R. rhizogenes* (step 22) in 100 µL total volume and spot onto plates of LB media supplemented with 50 µg/ml ALA (but no antibiotics) and then wait until the spot is dry 30m

24 Grow the biparental mating spot plates Overnight at 28 °C 16h

25 Scrape the biparental mating spot of *E. coli* ST18 + *R. rhizogenes* and resuspend in 1 mL sterile dH_2O — 5m

26 Centrifuge at 14000 rcf, 00:02:00 to pellet, and resuspend in 1 mL sterile dH_2O. This step should wash away any residual supplement from the mating plates that enables *E. coli* growth — 2m

27 Centrifuge again at 14000 rcf, 00:02:00 to pellet, and resuspend in 100 µL sterile dH_2O (The total volume will be more due to the pellet) — 2m

28 Transfer the resuspended mix from the previous step onto LB media plates supplemented with 100 µg/ml ampicillin, 50 µg/ml spectinomycin, and 100 µg/ml rifampicin. (No ALA). Plate out for single colonies — 2m

> **Note**
>
> For step 28: if the bacterial suspension is too thick then try diluting the resuspended mix by 1:10 in sterile water and plate this out in parallel

29 Incubate the plate(s) for 48:00:00 at 28 °C — 2d

30 Re-streak the *R. rhizogenes* strains carrying the construct of interest on LB agar containing 100 µg/ml ampicillin, 50 µg/ml spectinomycin, and 100 µg/ml rifampicin to ensure single colonies — 5m

31 Incubate the plate(s) for 48:00:00 at 28 °C — 2d

Preparation of seeds for hairy root transformation (for *Lotus j..*

32 To scarify the seeds, transfer the required number of seeds to a mortar and rub them with sand paper until they become white on the ends 2m

33 Transfer the seeds to a sterile tube (at least 15 ml capacity) and sterilise the seeds by immersing them in a 1% hypochlorite solution, and incubate in this solution for 15 min at Room temperature 15m

34 Remove the hypochlorite solution and discard appropriately. Add sterile water and invert the tube several times. Repeat this 5 times to wash the seeds and remove any residual hypochlorite 5m

35 Fill the tube with sterile water and incubate for at least 02:00:00 at Room temperature with shaking (alternativ incubate over night in coldroom) 2h

36 Using sterile forceps, transfer seeds to square petri dishes containing sterile filter paper soaked in sterile dH_2O (approximately 15 min per plate) 15m

37 Incubate the square plates containing the surface sterilised seeds sitting on damp filter paper from the previous step for 72:00:00 at 21 °C 3d

38 Transfer germinated seeds to a square petri dish that contains solid Gamborg B5 media including vitamins 15m

39 Grow seedlings for 72:00:00 at 21 °C with 16H/8H light/dark cycle, until the root has attached itself to the media 3d

Preparation of *R. rhizogenes* for hairy root transformation

40 Resuspend the *R. rhizogenes* strain from the agar plate (step 30-31) into sterile dH_2O as a thick 10m

suspension (OD_{600} >2). Approximately 100 µL is needed per plant, so make sure the volume of water for the suspension is sufficient for the number of plants that will be transformed

Note

The actual concentration is not important, there just needs to be an excess of the *R. rhizogenes*

Hairy root transformation of *Lotus* seedlings with *R. rhizogene..*

41 Wound seedlings with syringe needle (0.4mm) at the hypocotyl 1m

42 Add one large drop (~ 100 µL) of the thick suspension of *R. rhizogenes* (from step 40) on top of the wound 1m

43 Incubate the seedling for 01:00:00 horizontally to let the *R. rhizogenes* infect the hypocotyl 1h

44 Seal the plates containing the now infected seedlings with parafilm on the sides and bottom (prevents dehydration). Seal top with micropore tape on the top edge (Keeps the plates sealed but allows gas exchange) 1m

45 Grow the infected seedlings for 72:00:00 at 21 °C in the dark to enhance infection 3d

46 Grow the infected seedlings for 3 weeks at 21 °C with 16H/8H light/dark cycle until transformed roots emerge 3w

Selecting hairy roots

47 Hairy roots will emerge and develop from the infected wound sites

48 Place the plates containing the transformed plants on a transilluminator. Remove the non-fluorescently labelled roots (untransformed roots) using a scalpel 1h

Note

The Golden Gate assembly constructs should be designed to include a fluorescent marker to distinguish hairy roots from non-transformed roots. Alternative markers like RUBY can also be used

49 Transfer plants with transformed roots to new pots or plates and grow plants at 21 °C with 16H/8H light/dark cycle for one week 1w

For rhizobium inoculation of hairy roots (optional)

50 Resuspend the rhizobium strain (or your bacteria of interest) from a freshly streaked agar plate into sterile dH_2O 10m

51 Adjust the OD_{600} of the suspension to between 0.01 - 0.05 in a volume that is sufficient to provide 100 µL per plant to be inoculated 15m

51.1 If plants are on square agar plates: lay the square plate flat. Inoculate the roots of the plants by carefully applying 100 µL of the suspension from the previous step using a pipette. Ensure that you apply the inoculum evenly to as much of the root as possible 5m

51.2 Leave inoculated plant plate(s) flat for a short period so that the bacterial suspension can spread evenly across the plate and ensure contact with the roots 5m

51.3 If the plants are in pots: determine the volume that is equal to 100 µL X the number of plants present, and distribute this evenly to the plant pot substrate using a pipette 5m

52 Grow the inoculated plants for 3 weeks post inoculation at 21 °C with 16H/8H light/dark cycle until nodules are formed 3w
